# The "Bone of Contention"

**Published:** 1889-06-15

**Authors:** 


### THE "BONE OF CONTENTION."

The hospital system has been a "bone of contention" to many people for a long time. Hospital managers and officials are disposed to think that their institutions have been injured by the public criticism to which they have been subjected. That is entirely a mistake. If a thing is worth contending for it is of importance to the contenders; and the warmer the contention the stronger is the evidence of the value attached to the subject contended about. Have hospital managers so little faith in the merits of their charities that they fear to open the windows of their management and to let in the light of day? Surely not! The hospital system of the metropolis is a vast organisation, and it would be a very strange thing indeed if the prevalent microscopic criticism of the time did not here and there find a blot on its escutcheon. But what then? If we see a neighbour with a hole in his coat or a wart on his face, and tell him of it, do we therefore denounce the man himself as worthless, and a scoundrel? On the contrary, he may be a most excellent fellow, and our dearest friend. That is precisely the condition of certain hospital critics with regard to hospitals, as a whole. They may point out a fault here and there, but they regard the hospital system of this country, taken in its entirety, as one of the noblest developments of ancient or modern times.

This is an age of specialists and statistics; but no specialist can make statistics either popular or lovable. Those preachers and hearers who desire statistics for their guidance cannot do better than consult the tables published on another page, or, still better, the *Hospital Annual*, and form their own conclusions from the full and correct details therein set forth. But without the employment of a single figure or specific fact it is easy to make out a case for hospitals which places them absolutely at the head of all charitable institutions, for their public and private value to the community. For what is it that the hospitals of this country, and especially of the metropolis, are daily doing? Place on the one hand the various hospitals and their work, and on the other the cost of them all. Look first at the hospitals and their work. Is a low death-rate a sure indication of a high standard of general health? Has the death-rate in London and other large cities been steadily diminishing during the last twenty years? The practical knowledge obtained at hospitals and the scientific light radiated from them as from central suns have brought about that happy improvement in the public well-being. Have we in this country a class of eminent physicians, surgeons, and specialists, whose professional attainments are encyclopædic, whose trained skill is the admiration of the times, whose wisdom and honesty are relied upon with implicit confidence by the highest and the lowest in the land? Those attainments, that trained skill, that wisdom and severe honesty, are the direct product of the hospital wards. Have physiology and pathology illuminated some of the darkest recesses of human life and experience so that now the idiot is encouraged and educated, the lunatic is known to be the unfortunate victim of physical disease and treated accordingly, and even the weak-brained criminal is weighed in juster scales? All this has been made possible by hospitals, and by hospitals alone. Compared with the splendid work done by these institutions during the last fifty years, the cost, though admittedly great, is but as the small dust in the balance.

An enlightened self-interest, say some, is the most effective motor power for influencing men. That is as false in philosophy and in fact as it is base in conception. Nevertheless, it is clearly the intention of Providence that a man shall have a careful regard to his physical well-being and to the general health of the community. He who thinks the Divine Ruler will be pleased by unnecessary self-neglect has studied nature and revelation to little purpose. Does a man desire to do the best and the utmost that is in him for himself, his family, and the world? Then he must preserve his physical health at its highest level, and maintain his life to the greatest possible age. That which is true of the individual, is true of the whole body politic. Other things being equal, the people who are strongest and live longest will have sway and pre-eminence in the world's best work. Here it is seen that the highest self-interest coincides with the noblest duty. Do we wish to preserve our own health and that of the commonwealth? Then we shall do all that the occasion demands to preserve, to maintain, to strengthen, and to develop those great medical institutions which lie at the root and centre of health and prosperity, as the wholesome soil lies at the root of the trees, the flowers, and the corn. A practical people take action on practical facts. What facts are more self-evident and convincing than those made manifest every day in the life and work of hospitals? He who, being able to help them, refuses to do so, or helps them inadequately, pronounces a sorry verdict on his own intelligence and moral worth.

